# Shallow shotgun metagenomic sequencing of vaginal microbiomes with the Oxford Nanopore technology enables the reliable determination of vaginal community state types and broad community structures

**DOI:** 10.1186/s12866-025-04236-5

**Published:** 2025-08-25

**Authors:** Enid Graeber, Alona Tysha, Azlan Nisar, Daniel Wind, Werner Mendling, Patrick Finzer, Alexander Dilthey

**Affiliations:** 1 Institute of Medical Microbiology and Hospital Hygiene, Heinrich Heine University Düsseldorf, Düsseldorf, Germany; 2 German Center for Infections in Obstetrics and Gynecology, Wuppertal, Germany; 3 dus.ana - Düsseldorf Analytik, Düsseldorf, Germany; 4 Center for Digital Medicine, Heinrich Heine University Düsseldorf, Düsseldorf, Germany

**Keywords:** Sequencing, Oxford Nanopore Technologies, Vaginosis, Vaginal microbiome, Shallow shotgun metagenomic sequencing, Community state type, Illumina 16S

## Abstract

**Background:**

The vaginal microbiome plays an important role in female health; it is associated with reproductive success, susceptibility to sexually transmitted infections, and, importantly, the most prevalent vaginal condition in reproduction-age women, bacterial vaginosis (BV). Traditionally, 16S rRNA gene sequencing-based approaches have been used to characterize the composition of vaginal microbiomes, but shallow shotgun metagenomic sequencing (SMS) approaches, in particular when implemented with the Oxford Nanopore Technologies, have important potential advantages with respect to cost effectiveness, speed of data generation, and the availability of flexible multiplexing schemes.

**Results:**

Based on a study cohort of *n* = 52 women, of which 23 were diagnosed with BV, we evaluated the applicability of Nanopore-based SMS for the characterization of vaginal microbiomes in direct comparison to Illumina 16S-based sequencing. We observed perfect agreement between the two approaches with respect to detecting the dominance of individual samples by either Lactobacilli, vaginosis-associated, or other taxa; very high concordance (92%) with respect to community state type (CST) classification; and a high degree of concordance with respect to the overall clustering structures of the sequenced microbiomes. Comparing the inferred abundances of individual species in individual samples, we observed significant differences (Wilcoxon signed-rank test *p* < 0.05) between the two approaches for 12 of the 20 species most abundant in our cohort, indicating differences in the fine-scale characterization of vaginal microbiomes. Higher overall abundance of *Gardnerella vaginalis*, associated with an increased number of CST IV detections, in the Nanopore shallow SMS data indicated potentially increased sensitivity of this approach to dysbiotic states of the vaginal microbiome. Nanopore shallow SMS also enabled the methylation-based quantification of different human cell types in the characterized samples as well as the detection of non-prokaryotic species, including *Lactobacillus* phage and *Candida albicans* in study participants with microscopically detected *Candida*. One important potential limitation of the evaluated Nanopore-based SMS approach was marked variation in sequencing yields.

**Conclusion:**

Our study demonstrated the successful application and potential advantages of Nanopore-based shallow SMS for the characterization of vaginal microbiomes and paves the way for its application in larger-scale research or diagnostic settings.

**Supplementary Information:**

The online version contains supplementary material available at 10.1186/s12866-025-04236-5.

## Background

The human vagina is colonized by a characteristic microbial community [1, 2]. In reproductive-age women, the vaginal microbiome is typically dominated by a single *Lactobacillus* species or comprised of a more diverse community of facultative and obligate anaerobic bacterial species [3]. Vaginal microbiome community structures are often categorized using the Community State Type (CST) system [3, 4]; CSTs I, II, III and V are defined by dominance of, respectively, *Lactobacillus crispatus*, *Lactobacillus gasseri*, *Lactobacillus iners* and *Lactobacillus jensenii*; CST IV, by contrast, captures diverse vaginal microbiomes characterized by a mixture of anaerobes. Domination of the vaginal microbiome by lactobacilli is generally associated with favourable health outcomes, for example with respect to reproduction [5–7] or susceptibility to sexually transmitted diseases [8, 9]. Importantly, the “diverse” CST IV is also associated with symptomatic bacterial vaginosis (BV) [10, 11], a highly prevalent and often refractory condition [10, 12, 13] associated with severe reductions in subjective wellbeing [14] and an increased likelihood of preterm birth [15–19]. While the precise aetiology of BV is still unclear, it is often understood to represent a dysbiotic state of the vaginal microbiome [20]; of note, however, many women with CST IV vaginal microbiomes do not show symptoms of BV, and the observed associations between CST IV and BV differ between ethnicities [3].

Most approaches for characterizing the vaginal microbiome are based on 16S sequencing, targeting, by polymerase chain reaction (PCR), either the 16S rRNA gene in its entirety (which is only possible with long-read technologies such as Oxford Nanopore [21, 22]; or specific “variable regions”, located between more conserved regions of the 16S gene [3, 23, 24]. For vaginal microbiomes, targeting the V1-V2 region of the 16S gene has been applied successfully to identify and characterize the community state types (CSTs) by Ravel et al. [3]. It is well-known, however, that the choice of target variable regions can impact measured community compositions [25–27]; in general, amplification-based measures of community composition cannot be assumed to represent true biological abundances of different organisms [28, 29]. Furthermore, 16S sequencing is limited to characterizing the prokaryotic component of the microbiome.

Shotgun metagenomic sequencing (SMS) has therefore emerged as an attractive approach for characterizing the vaginal microbiome [30–33]. In contrast to 16S-based methods, SMS approaches do not require amplification and enable the detection and relative quantification of DNA viruses and eukaryotes [34–36] including the fungal component of the microbiome, which is known to play an important role in vaginal microbial communities [37, 38]. Challenges in the context of SMS-based microbiome characterization [39], however, include a high fraction of “contaminant” host DNA; higher requirements with respect to the quantity and quality of input DNA, which may, depending on sampling approach and study design, not always be easy to satisfy; and increased per-sample sequencing costs when compared to 16S-based approaches, at least when applying high-coverage SMS approaches.

Shallow shotgun metagenomic sequencing [33, 40, 41], i.e., shallow SMS with a reduced per-sample sequencing depths, exhibits reduced per-sample sequencing costs and may thus enable the cost-effective metagenomic characterization of vaginal microbiome samples while maintaining many of the advantages of SMS for research or diagnostic settings. Furthermore, while SMS is generally sequencing technology-agnostic, the Oxford Nanopore platform may be particularly suitable for the implementation of shallow SMS due to the availability of flexible flow cell and multiplexing schemes that enable the cost-effective generation of relatively low amounts of sequencing data, e.g. from a single Flongle flow cell without multiplexing to standard Flow Cells with up to 96-sample multiplexing. An additional advantage of the Nanopore technology includes rapid “real-time” data generation. However, the ability of shallow SMS in general and Nanopore-based shallow SMS in particular to accurately characterize important properties of the vaginal microbiome, such as CSTs, has not been evaluated yet.

We thus set out to conduct a pilot study to evaluate the applicability of Nanopore-based shallow SMS for the characterization of vaginal microbiomes in direct comparison to an established Illumina 16S-based approach, including samples from individuals with *Lactobacillus*-dominated vaginal microbiomes as well as from individuals diagnosed with BV. In addition to benchmarking the performance of Nanopore-based shallow SMS against the established Illumina approach, we also explore the characterization of sample features not accessible to Illumina 16S, such as the proportion of host DNA; the presence of non-prokaryotic species; and methylation patterns of host DNA.

## Methods

### Study cohort

Study participants were recruited between February 2020 and May 2021 from patients seeking care at the German Center for Infections in Gynecology and Obstetrics, located at Helios Hospital, Wuppertal, Germany. Participation in the study was offered after an initial screening for putative presence of BV, aiming to include approximately 25 patients with BV and 25 without BV.

All study participants underwent detailed clinical examination and anamnestic assessment; data collected for the study included age, height, weight, BMI, smoking status, ethnicity, contraception status, relationship status, number of births and children, history of relevant diseases, the presence of BV according to Amsel criteria [42], and the presence of other relevant clinical indications. The vaginal pH value was determined directly during the examination and a wet mount was prepared and assessed under the microscope. Afterwards the wet mount was Gram stained to determine the Nugent score (Nugent et al. 1991). In addition, vaginal smears were collected in two 2 mL ZymoBIOMICS DNA/RNA Shield Collection Tubes (USA) and stored directly; they were used for microbiome sequencing.

### DNA extraction

ZymoBIOMICS DNA/RNA Miniprep Kit (cat. Nr. R2002) was used to extract DNA from the ZymoBIOMICS DNA/RNA Shield Collection solution, according to v.2.0.0 of the user manual from 2020/9/10 with following modifications. Samples were resuspended by vortexing and 200 μL of suspension were transferred into the beat beating tube. An additional 350 μL of DNA/RNA Shield buffer were added to enable the harvesting 200 µL of bead-free liquid. Bead beating was performed using Vortex genie v2 pulse with 24 multi-tube attachment on maximal speed for 40 min. Further steps were conducted according to part IV of the user manual with an elution step in 100 µL of nuclease-free water and using the Qubit 3 device (Qubit 1× dsDNA HS Assay Kit). For all study participants, two extractions were attempted and the extraction used for sequencing was determined based on the amount of extracted DNA. In some instances where the extracted amount of DNA was below 1 ng/µL, a third extraction was carried out; if DNA could not be extracted successfully, the sample was excluded. Illumina and Nanopore sequencing data were always generated from the same extraction for each study participant; first, 10 ng of DNA in 10 µL were taken off for Illumina 16S sequencing, and the rest was used for Nanopore SMS.

### Illumina 16S sequencing

Illumina 16S library preparation was conducted following the manufacturer’s protocol, utilising the QIAseq 16S/ITS Panel with V1–V2 and V2–V3 16S primers (Qiagen) and with a total input of 1 μL per sample, as recommended for samples with low input. Libraries were normalised to 4 nM, pooled, and subsequently sequenced on a MiSeq system (Illumina Inc.) with a read setup of 2 × 301 bp and 20% PhiX addition.

### Oxford Nanopore metagenomic sequencing

Library preparation of Oxford Nanopore metagenomic sequencing was carried out with the ligation sequencing kit DNA SQK-LSK109 (version NBE_9065_v109_revAH_14Aug2019); barcoding (12–16 samples per flow cell) was based on the EXP-NBD196 expansion kit. Short fragment buffer (SFB) was used in the adapter ligation step to ensure equal purification of short and long DNA fragments. The resulting library was sequenced on Nanopore GridION with R9.4.1 flow cells (type FLO-MIN106). Basecalling and demultiplexing were carried out using MinKNOW (v. 21.11.6) with MinKNOW Core (v. 4.5.4) and Guppy (v. 5.1.12).

### Data analysis

#### Analysis of Illumina 16S sequencing data

Illumina 16S data were analysed with Emu v. 2.0.1 [22] in short-read mode with the default reference database, yielding species-level relative abundances (refer to the Emu command in Supplementary file S1). Results from the Emu analysis were used in subsequent analyses without normalization for 16S rRNA gene copy number variation among taxa.

#### Analysis of Nanopore SMS data

Nanopore SMS reads were analysed with Kraken2 [43] (v. 2.1.1) followed by Bracken [44] (v. 2.5) at the “species” level (Supplementary file S1). The database used for the Kraken2/Bracken analyses comprised 29,443 genomes from RefSeq classified as “complete” or “reference” belonging to divisions bacteria, fungi, protozoa and viruses and three well-characterized human references [45, 46] (Supplementary table S2). For comparing the relative abundances based on Nanopore SMS and Illumina 16S sequencing, Bracken-based prokaryotic abundances were calculated by dividing read counts of prokaryotic species by the total number of prokaryotic reads (classified to superkingdom “Bacteria”, NCBI taxonomy ID 2).

#### CST classification and calculation of Shannon diversity

CST classification was carried out using a custom script according to the following criterion: If a sample had a dominating species (> 50% abundance) of *L. crispatus*, *L. gasseri*, *L. iners*, or *L. jensenii*, the sample was assigned a CST of I, II, III, or V, respectively. If a sample had no dominating species or if the dominating species was an anaerobic strain other than *Lactobacillus*, the sample was assigned CST IV. The Shannon diversity index was calculated based on relative bacterial abundances in a custom Python script as the negative sum of the products of each nonzero abundance and its natural logarithm.

#### Principal coordinates analysis

Principal coordinates analysis (PCoA) was performed using the skbio.stats.ordination.pcoa method from scikit-bio library v.0.5.6 [47]. Bray-Curtis dissimilarity was calculated for each sample pair based on vectors of relative species abundances. The resulting Bray-Curtis dissimilarity matrix was used as an input for PCoA analysis. A kernel density estimate (KDE) plot was added to the generated visualisations to highlight the different densities of data clusters. KDE plots were generated using the “kdeplot” method from Python Seaborn package v.0.2.12 [48] with the package’s default Gaussian kernel and standard deviation of the smoothing kernel. The number of contour levels was set to 10 with levels = 10 option, each representing a region of equal estimated density of the data points.

#### Statistical testing

Statistical analysis of the data was performed on several stages using scipy.stats module v.1.10.0 [49] in Python. For details refer to Supplementary file S1. Assuming the samples in the cohort were independent, cohort and sequencing metrics were compared using the Mann-Whitney U test (scipy.stats.mannwhitneyu). Samples derived from the same biological specimen and analysed using two different sequencing techniques were treated as paired. Pearson’s correlation was computed using scipy.stats.pearsonr, and the Wilcoxon signed-rank test was performed using scipy.stats.wilcoxon. Hodges-Lehmann estimate of location shift for paired samples was calculated according to [50].

#### Data visualization

Visualizations were generated using custom Python scripts with the Seaborn [48] v. 0.12.2 and Matplotlib [51] (matplotlib-base v. 3.6.2) packages. Relative species abundances were visualized as a clustered heatmap using the seaborn.clustermap module. Patient sample columns were clustered using UPGMA linkage based on Euclidean distances. Statistical significance annotations on boxplots were added using statannotations [52] v. 0.6.0. Pairwise statistical comparisons were performed based on the Mann–Whitney U test, and significance levels were indicated using standard notation.

#### Verification of eukaryotic and viral species in Nanopore SMS data

Non-human eukaryotic and viral species reported by Bracken [44] were validated on a read level to ensure the accuracy of classification using a two-level strategy. As Bracken does not support read-level classification, reads assigned by Kraken2 [43] to the species of interest were identified and extracted. For the first-level filter, the extracted reads for each target species were aligned with Minimap2 [53] to the set of species-specific reference genomes in the utilized Kraken2 database. For each species, we determined (i) the proportion of extracted reads mapping to any of the utilized species-specific reference genomes; (ii) the median proportion of read bases covered by the primary alignments, based on the alignments’ CIGAR strings; (iii) the median mismatch rate across primary alignments, calculated for each alignment by dividing the NM value by the alignment length. To pass the first-level filter, we required a proportion of aligned reads of at least 50%, a median alignment coverage of at least 50%, and an alignment error rate of $\:\le\:$13%, pragmatically set based on a typical within-species ANI divergence of up to 5% [54] plus a Nanopore R9.4 sequencing error rate of up to 8% for bacterial genomes [55]. All species that did not pass the first-level filter were classified as not validated. For the second-level filter, we selected the best single reference genome for each species based on the number of read alignments generated during the first filtering step. For each species, we re-aligned the set of extracted reads against the selected reference genome and visually assessed the evenness of read distribution along the genome; species that exhibited an aberrant distribution of reads were classified as not validated. To confirm the species validation results, we carried out an additional BLASTn database search (nr/nt; from 28.11.2022) for all Kraken2-extracted reads, allowing for up to five hits from five different genomes (see Supplementary file S1). In all instances in which a species was classified as validated according to the two-level filter strategy (see above), assignment of > 95% of the extracted reads to the species was also confirmed by BLASTn.

#### Methylation-aware Nanopore basecalling and cell-type-of-origin analysis

Methylation-aware basecalling of human Nanopore sequencing reads was carried out with Guppy 6.5.7 (config module: dna_r9.4.1_450bps_modbases_5mc_cg_hac.cfg; reference genome: GRCh37 hg19 b37 [56]), producing BAM files with modification calls in MM and ML tags. Sequencing read statistics (average coverage, number of methylated reads, number of total reads, and number of total mapped reads) were computed using a custom bash script and Samtools v.1.18 [57]. Modkit 0.2.7 [58] with default parameters was used to convert the information from the BAM files into tab-separated BED files, specifying the number of modified and non-modified bases at each CpG position; post-processing of this file was carried out in R 4.4.0 [59]. Positions in the BED file that were also covered by the Illumina Infinium 450k methylation array were identified and mapped onto Infinium 450k probes using a custom R script from [60], producing a file specifying a methylation score for each Infinium 450k probe based on the generated Nanopore sequencing reads. Cell type deconvolution was carried out using non-negative least squares regression [61–63], using code available at [64], including 1000 hypermethylated and hypomethylated CpGs for 25 cell types as input. The reference atlas used for the deconvolution process was generated using the script [65], with the input parameter being set to 1000, and plots were created using [66]. Post-deconvolution analyses were limited to samples with read coverage $\:\ge\:$ 0.2X on the human genome; this threshold was chosen based on simulations that demonstrated, consistent with the literature [62] robust cell type deconvolution from 0.2X coverage (data not shown), as well as based on visual inspection of per-sample coverage. Tissue composition differences between non-BV and BV groups were assessed using the Mann–Whitney U test. Analyses were conducted with scipy.stats.mannwhitneyu function of scipy.stats module v.1.10.0 [49] in Python.

## Results

### Recruitment of a study cohort

We recruited a study cohort of 56 women of mainly European ancestry. For 52 study participants, DNA extraction from vaginal smears was successful; the other 4 study participants and their samples were excluded from all analyses. 44% (*n* = 23/52) of the included study participants had BV; additional diagnoses present in the cohort included vulvodynia and Lichen sclerosus. There were no significant differences between BV and non-BV groups with respect to age, BMI and number of children (Supplementary table S3); as expected, study participants with BV had a higher Nugent score (mean BV: 6.7 vs. non-BV: 0.7, Mann-Whitney U *p* = 7.1e−9) and higher vaginal pH (mean BV: 5.1 vs. non-BV: 4.2, Mann–Whitney U *p* = 2.2e−5) than study participants without BV. Nugent scores did not always align with clinically determined Amsel criteria; six of 23 included BV-positive participants had Nugent scores below 7.

### Baseline characterization of the cohort with Illumina 16S sequencing

We carried out a baseline characterization of vaginal microbiomes in our study cohort based on Illumina 16S sequencing. Illumina 16S sequencing data were successfully generated for all 52 study participants included after successful DNA extraction; sequencing depths ranged from 144,589 to 295,795 reads per sample (Supplementary table S4), with the BV group exhibiting an increased number of sequenced reads (mean 236,082 compared to 191,279 for the non-BV group; Mann-Whitney U *p* = 2.5e−5; Supplementary figure S5). 94% of samples had a read classification rate of ≥ 85% (Supplementary table S4).

**Fig. 1:**
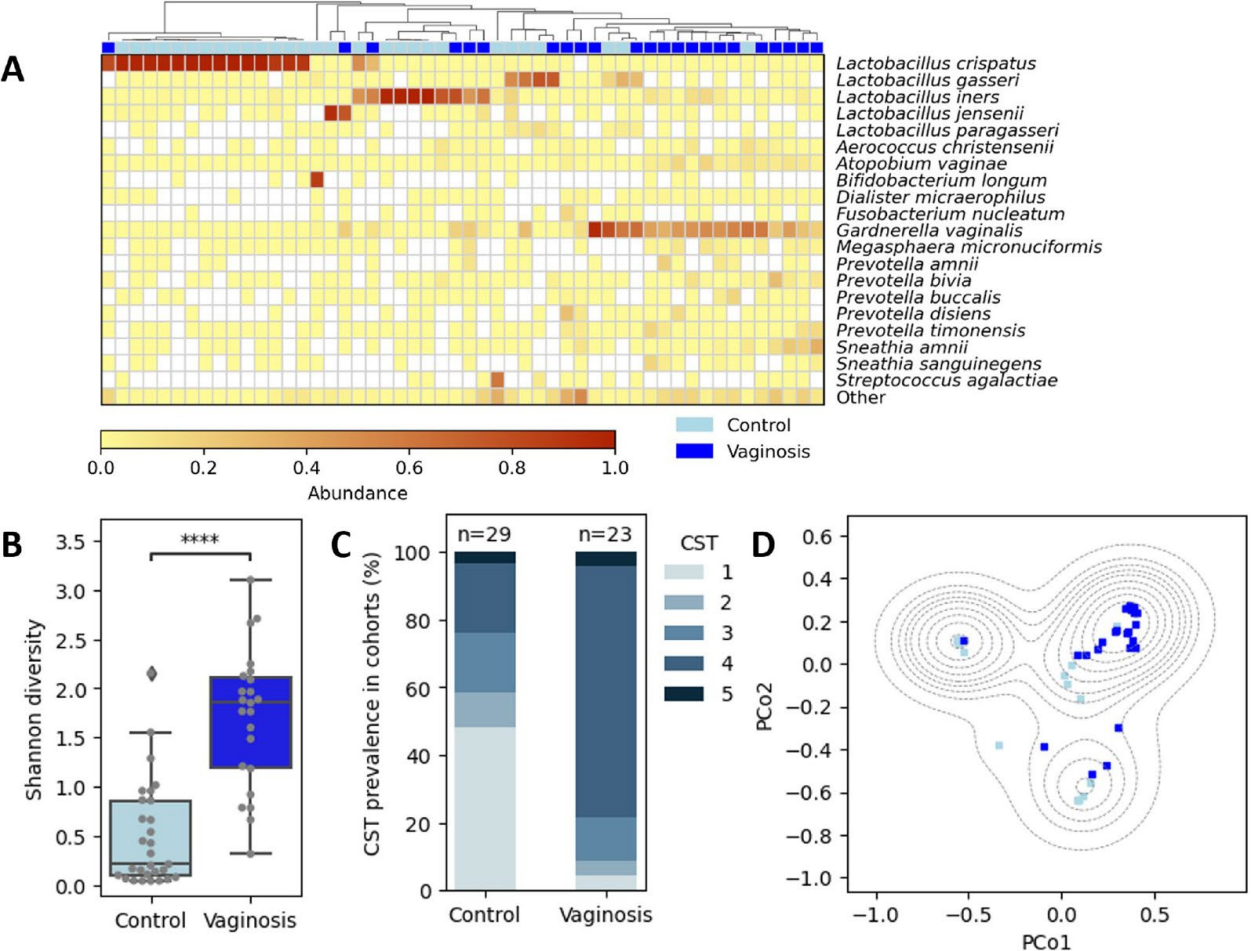
Illumina 16S-based characterization of the cohort. **A** Illumina 16S-based relative abundances; shown are relative abundances of the 20 most abundant species (cumulative abundance across all samples) as well as the cumulative abundance of all other species (“Other”). Filled squares above the heatmap indicate the BV status of study participants (dark blue = BV; light blue = non-BV). Columns are clustered based on Euclidean distances. **B** Shannon diversity for BV (“Vaginosis”) and non-BV (“Control”) groups after rarefaction to 140,000 reads per sample. **C** Community State Type (CST) distribution for BV (“Vaginosis”) and non-BV (“Control”) groups. **D** Principal Coordinates Analysis (PCoA) based on Illumina 16S sequencing and Bray-Curtis inter-sample distances. Dots are colored according to BV status (dark blue = BV; light blue = non-BV). Also shown is a contour plot of the probability density function (KDE) in dashed lines, which represent areas of equal probability density

As expected, most (*n* = 21) of the non-BV samples were dominated by one of four *Lactobacillus* species. In contrast, most BV samples exhibited high abundances of *Gardnerella vaginalis* and anaerobic bacteria of the genera *Prevotella*, *Sneathia*, *Megasphaera*, *Atopobium*, *Dialister* and *Porphyromonas* (Fig. 1A, Supplementary table S6), which are commonly associated with vaginosis [19, 67]; in addition, a small number of BV samples were dominated by *L. iners* or *L. gasseri*, also in accordance with the literature [68]. BV samples showed significantly higher microbial diversity compared to control samples after rarefaction at a depth of 140,000 reads (mean Shannon index 1.7 in the BV group compared to 0.49 in the non-BV group; Mann-Whitney U p-value = 6.5e-7; Fig. 1B), ensuring that diversity estimates were not driven by differences in sequencing depth (Supplementary figure S7).

We carried out CST classification for each sample (Supplementary table S6); in the non-BV group, CSTs I (48%; *n* = 14), IV (21%; *n* = 6), and III (17%; *n* = 5) were most frequently observed, whereas CST IV (74%; *n* = 17) was most prevalent in the BV group (Fig. 1C).

To further investigate the structure of the sequenced vaginal microbiomes, we carried out Principal Coordinates Analysis (PCoA) based on Bray-Curtis distances and observed a three-cluster structure (Fig. 1D), corresponding to microbiome domination by *L. crispatus* (first cluster; CST I; 15 samples); a high abundance ($\:\ge\:$40%) of *L. iners* (second cluster; CSTs III and IV with 8 and 2 samples, respectively); and diverse vaginal microbiome structures (third cluster; CSTs II, V and IV with 4, 2, and 21 samples, respectively). The third cluster contained the large majority (*n* = 18/23) of BV samples; 4 BV samples were part of the second cluster; and one BV sample (ID = 34) was part of the first cluster corresponding to CST I. In addition to domination by *L. crispatus* (relative abundance 80.6%), sample 34 also had a relatively high abundance of *Bifidobacterium dentium*, a vaginal pH of 4.2, and a Nugent score of 0, suggesting a transitory situation between BV and a healthy status [68] or a potential false-positive classification of sample 34 as BV-positive.

In conclusion, the vaginal microbiomes in our cohort were broadly in line with microbiome structures expected for women with and without BV.

### Evaluation of Nanopore shallow SMS for the determination of vaginal community state types and broad-scale community structures

We proceeded to evaluate the applicability of Nanopore shallow SMS for the characterization of vaginal microbiomes. For each sample, Nanopore sequencing data were generated from the same DNA extraction that was also used for Illumina 16S sequencing, and we carried out a direct comparison between the two methods with respect to community composition and CST classification.

Per-sample Nanopore sequencing depths (Supplementary table S4) exhibited a larger variation than Illumina sequencing depths, ranging from 830 to 1,689,317 reads per sample (mean = 379,988); no significant difference was observed for the average number of reads in the BV and non-BV groups (mean read depths = 493,760 and 289,755, respectively; Mann-Whitney U p-value = 8.7e−2; Supplementary figure S5). The generated Nanopore sequencing reads exhibited a mean classification rate of 98.4%, with almost all (*n* = 51/52) samples exhibiting a classification rate $\:\ge\:$95%. The large majority ($\:\ge\:$90%) of classified reads were human in almost all samples (*n* = 49/51), with the relative abundance of prokaryotic reads among the classified reads ranging from 0.1 to 16.6% (mean 2.9%), and absolute prokaryotic read counts ranging from 11 to 79,272 (mean = 9,688). To account for increased variance in community composition estimates in samples with low read counts, we pragmatically set a threshold of ≥ 200 prokaryotic reads for all further analyses of the vaginal microbiome based on shallow SMS; this led to the exclusion of samples 24 and 29 with 58 and 11 prokaryotic reads, respectively (Supplementary table S8).

**Fig. 2:**
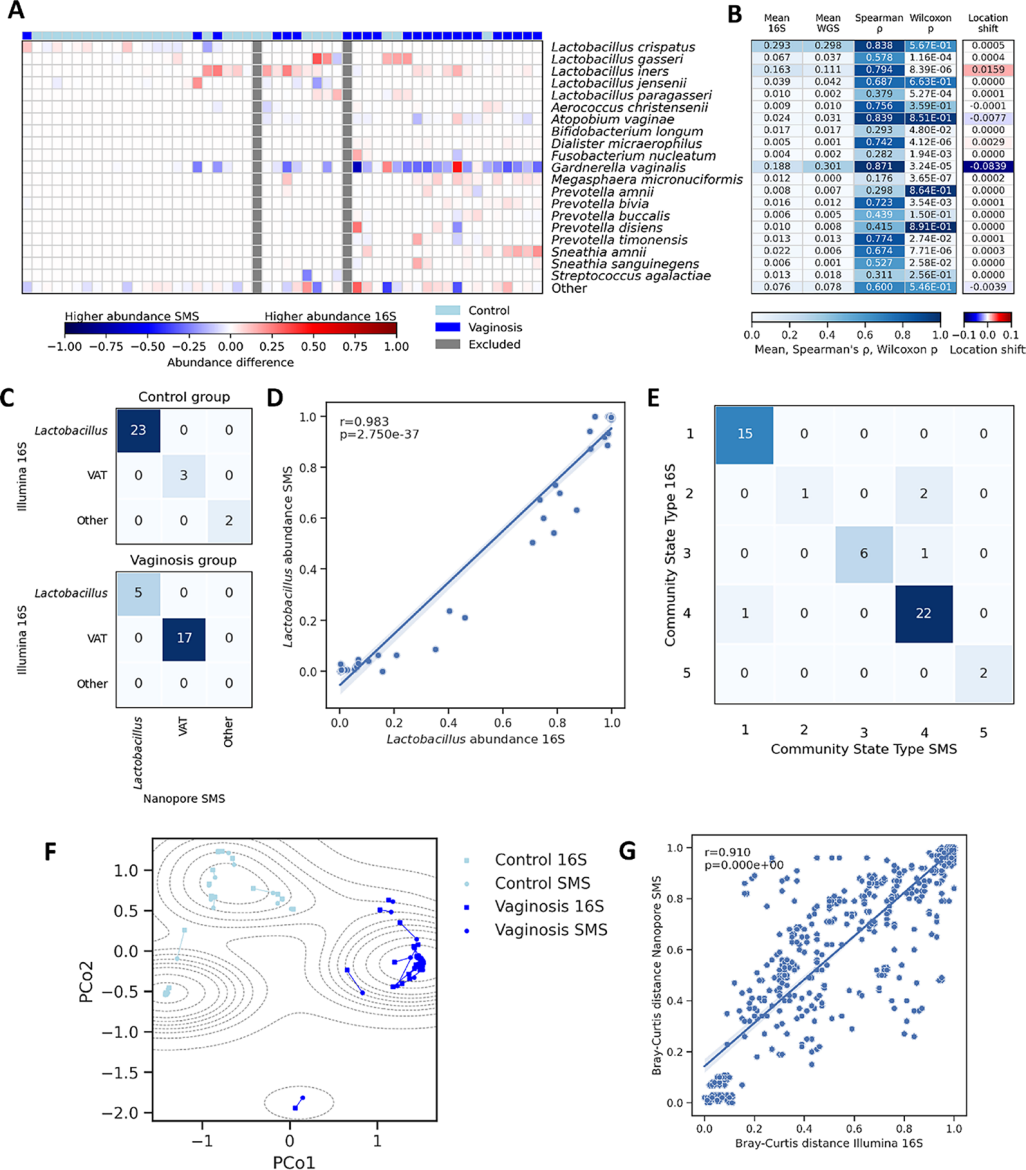
Comparison of Nanopore shallow SMS and Illumina 16S assessments of vaginal bacterial communities. **A** Species-level visualization of the per-sample differences between Nanopore- and Illumina-based abundance estimates, shown for the 20 species exhibiting the highest cumulative abundance across all samples in Illumina 16S data; the “Other” category shows the combined abundance of the remaining species. Two samples were excluded because their prokaryotic read count in the Nanopore data was below the utilized threshold of 200. BV status is indicated by filled squares above the heatmap (dark blue = BV; light blue = non-BV). Columns are arranged in the same order as in Fig. 1A. **B** Statistical comparison of Nanopore- and Illumina-based abundances of individual species for the species from panel **A**. Shown are, for each species, mean relative abundance in Nanopore and Illumina data, as well as Spearaman's correlation, Wilcoxon signed-rank p-value, and Hodges-Lehmann estimate of location shift computed for the Nanopore- and Illumina-based abundances of the included species in individual samples. **C** Confusion matrix for detecting the domination (defined as relative abundance ≥ 50%) of individual samples by lactobacilli, vaginosis-associated taxa (VAT), or other taxa (see main text for definitions). Results are based on Nanopore shallow shotgun metagenomic sequencing (SMS) and Illumina 16S data, for the 50 samples with a prokaryotic read count exceeding the threshold of 200. **D** Nanopore- and Illumina-based cumulative abundances of *Lactobacillus* species in individual samples; also shown are Pearson’s r, the corresponding p-value, and a least-squares linear regression line with intercept. **E** Confusion matrix for detecting the Community State Type (CST) of individual samples based on Nanopore shallow SMS and Illumina 16S; included are the 50 samples with a prokaryotic read count exceeding the utilized threshold of 200. **F** Joint Principal Coordinates Analysis (PCoA) of Nanopore shallow SMS- and Illumina 16S-based microbiome composition vectors; each biological sample is represented by two dots connected with a line. Also shown is a contour plot of the probability density function (KDE) in dashed lines, which represent areas of equal probability density. **G** Comparison of Nanopore- and Illumina-based Bray-Curtis inter-sample distances for all 1225 unique pairs of biological samples. Also shown are Pearson’s r, the corresponding p-value, and a least-squares linear regression line

First, we evaluated concordance between Nanopore-based shallow SMS and Illumina 16S with respect to large-scale community structure. We found perfect agreement between Nanopore-based shallow SMS and Illumina 16S with respect to dominance (defined as relative abundance ≥ 50%) of individual samples by either lactobacilli, vaginosis-associated taxa (defined based on [19] and [67]: *G. vaginalis* and anaerobic bacterial genera *Prevotella*, *Sneathia*, *Megasphaera*, *Atopobium*, *Dialister*, and *Porphyromonas*), or other taxa (Fig. 2C). The relative cumulative abundance of *Lactobacillus* species was highly correlated (Pearson’s *r* = 0.983; *p* = 2.7e−37; Fig. 2D) between Illumina 16S and Nanopore-based shallow SMS, and high correlations, ranging from 0.904 for *L. gasseri* to 0.996 for *L. crispatus*, were observed for the 4 most abundant individual *Lactobacillus* species (Supplementary figure S9).

Second, we carried out CST classification for each sample based on the Nanopore shallow SMS data and observed concordance with Illumina 16S-based CSTs for 92% (*n* = 46/50) of samples (Fig. 2E). All 4 observed discrepancies were associated with CST IV, with 3 detections of CST IV exclusive to the Nanopore data and 1 detection exclusive to the Illumina 16S data. Further investigation of these discrepancies showed that they were driven by shifts in relative abundances of the same species, rather than changes in the presence of distinct taxa (Supplementary tables S6 and S8); for example, in sample 17, Illumina 16S analysis showed relative abundances of 28% for *G. vaginalis* and 61% for *L. gasseri*, whereas the Nanopore-based analysis showed relative abundances of 48% for *G. vaginalis* and 40% for *L. gasseri*.

Third, to characterize differences between the two technologies at the level of overall community composition, we first performed PCoA based on the Nanopore data and found that the Nanopore-based PCoA recovered the structure observed in the Illumina-based PCoA (one compact cluster dominated by *L. crispatus*; a second cluster dominated by *L. iners*; and a third cluster representing the remaining samples; Supplementary figure S10). In addition, we carried out a joint PCoA (i.e., based on a composition matrix in which each biological sample was represented by two separate entries, corresponding to the Illumina- and Nanopore-based composition vectors, respectively), and observed a tight clustering between Illumina- and Nanopore-based estimated compositions for individual samples (Fig. 2F). Last, we found a significant correlation (Pearson’s *r* = 0.91, *p* = 0.0) between Illumina- and Nanopore-derived inter-sample Bray-Curtis distances computed for all *n* = 1225 unique pairs of biological samples (Fig. 2G). In conclusion, we found that overall community compositions determined by Illumina 16S and Nanopore SMS were broadly similar, but not identical.

Fourth, we evaluated the agreement between Nanopore SMS and Illumina 16S at the level of individual species (Fig. 2A). For this comparison, we focused on the 20 most abundant species in our cohort (cumulative abundance across all samples) and assessed the degree of concordance between the two sequencing approaches with 4 different metrics (mean relative abundance of the species across at the complete-cohort level, as well as Spearman's correlation, Wilcoxon signed-rank significance, and Hodges-Lehmann estimates for individual-level species abundances; Fig. 2B). Based on Spearman's correlation, we observed a relatively high degree of correlation between Nanopore SMS and Illumina 16S-based relative abundances (Spearman’s ρ > 0.7) for 8 of 20 species. Based on the paired Wilcoxon signed-rank test, we observed significant differences in estimated per-sample relative abundances (Wilcoxon signed-rank test *p* < 0.05) for 12/20 species, indicating that these species were systematically either overestimated or underestimated by one of the methods. Of note, 7 of the 12 species that had a correlation < 0.7 were also detected as exhibiting a significant difference in estimated per-sample relative abundances (*L. gasseri; Lactobacillus paragasseri*; *Bifidobacterium longum*; *Fusobacterium nucleatum*; *Megasphaera micronuciformis*; *Sneathia amnii; Sneathia sanguinegens*). Out of the 12 species for which we observed a significant difference in per-sample relative abundances (Wilcoxon signed-rank test *p* < 0.05), 10 had relatively low (≤ 10%) mean abundances across the cohort according to both Illumina 16S and Nanopore shallow SMS; *L. iners* (Illumina- and Nanopore-based relative abundances of 16% and 11%, respectively) and *G. vaginalis* (Illumina- and Nanopore-based mean relative abundances of 19% and 30%, respectively) were the two exceptions. Relative location shifts for these two species (0.02 for *L. iners* and − 0.08 for *G. vaginalis)* indicated higher and lower abundances in the Illumina 16S data for *L. iners* and *G. vaginalis*, respectively; furthermore, *G. vaginalis* was the only species that had a significant Wilcoxon signed-rank p-value and an estimated negative location shift, i.e. indicating systematically increased abundances in the Nanopore SMS data.

### Detection of non-prokaryotic species and human methylation patterns based on Nanopore SMS

**Fig. 3:**
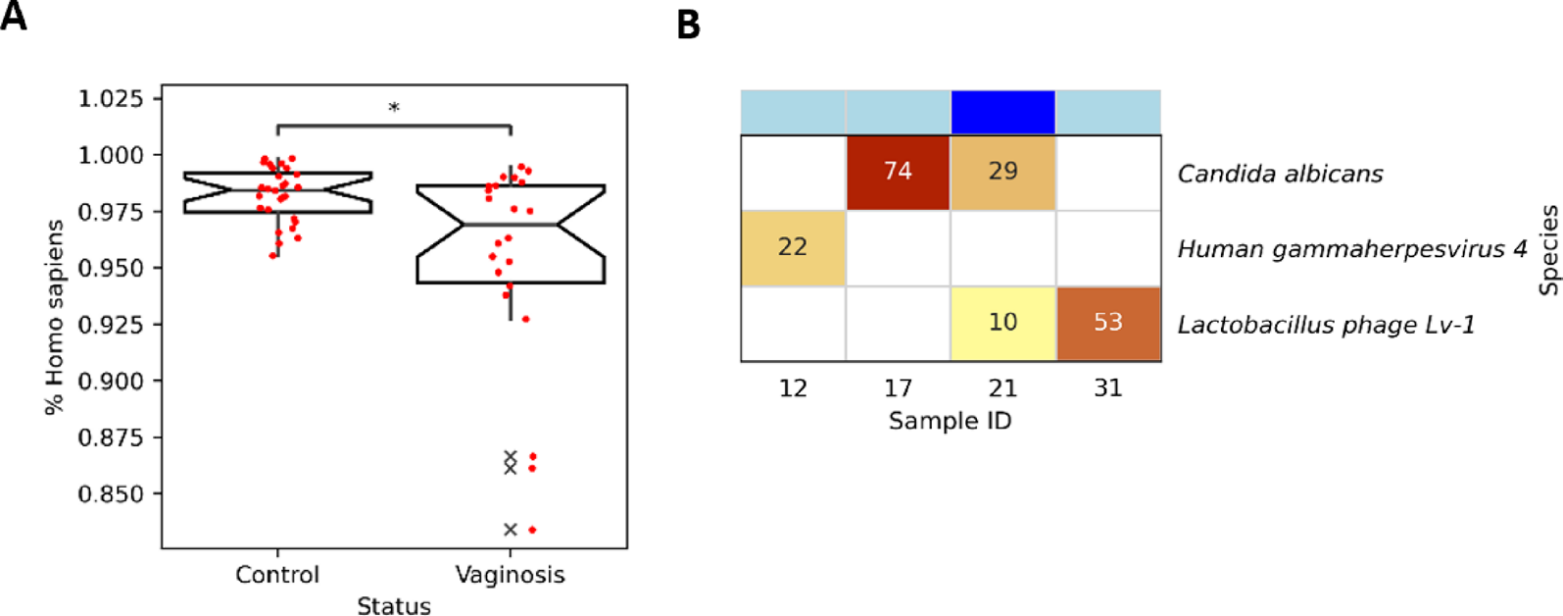
Human read fraction and presence of non-prokaryotic species determined based on Nanopore shallow SMS. **A** Fraction of human reads for BV (“Vaginosis”) and non-BV (“Control”) groups; significance was determined with the Mann–Whitney U test and a jitter plot of the distribution of the human reads fraction in the two groups is also shown. **B** Absolute read counts for detected non-human eukaryotic and viral species; only species detections that passed all verification steps (see text) are included. BV status of the included samples is indicated by filled squares above the heatmap (dark blue = BV; light blue = non-BV)

We carried out an exploratory analysis of microbiome features that could be characterized by Nanopore shallow SMS, but not by Illumina 16S. First, Nanopore shallow SMS could enable a quantification of the proportion of host DNA. In our cohort, between 83.4% and 99.8% of classified Nanopore SMS reads were of human origin (Fig. 3A, Supplementary table S4), but we found statistically significant difference between the BV and non-BV groups (mean BV: 95.4% vs. non-BV: 98.2%, Mann-Whitney U *p* = 1.3e−02). Second, Nanopore shallow SMS may enable the detection of non-prokaryotic species. Initial read classifications by Bracken comprised 112 individual detections of non-human eukaryotic and viral species (3 different viral species across 4 samples and 30 eukaryotic species across 16 samples; Supplementary table S11); reliable metagenomic detection of non-prokaryotic species, however, is well-known to be a challenging problem [34], in particular from low numbers of reads [69]. Employing a multi-level verification approach based on read back-mapping, assessing uniformity of reference genome coverage, and BLASTn (see Methods) reduced the number of detections from 113 to 5; 28 eukaryotic species failed the back-mapping step, and all remaining *T. gondii* detections as well as one detection of *Lactobacillus prophage Lj771* failed visual inspection (see Supplementary figure S12 for example read distributions plots). Overall, we established the presence of two viral species (*Lactobacillus phage Lv-1*, *Human gammaherpesvirus 4*) across 3 samples and of one eukaryotic species (*Candida albicans*) across 2 samples (one of which also contained *Lactobacillus phage Lv-1*; Fig. 3B). Of note, for individuals 17 and 21, for which we detected the presence of *Candida albicans* based on Nanopore shallow SMS, the presence of *Candida* was also detected microscopically. Third, based on the analysis of DNA methylation, Nanopore sequencing may enable a quantitative characterization of human cell types present in the vaginal epithelium. In a subset of *n* = 17/52 samples with $\:\ge\:$ 0.2X average coverage of the human genome (Supplementary figure S13) and based on a tissue-deconvolution approach (see Methods), we determined the human cell type compositions of our study samples. The three cell types with the highest cumulative abundances were neutrophils, head and neck laryngeal cells, and prostate cells (Supplementary table S14); no significant differences (threshold *p* = 0.05), however, were observed for individual cell type relative abundances between the BV and non-BV groups.

## Discussion

Nanopore-based shallow SMS may enable the generation of vaginal microbiome sequencing data in a more cost-effective, more rapid, and more flexible (with respect to the availability of multiplexing schemes) manner than Illumina 16S sequencing-based approaches; in addition, it may also enable the characterization of microbiome features not accessible to 16S sequencing. Based on a study cohort of *n* = 52 women, of whom *n* = 23 were diagnosed with BV by at least 3 of 4 Amsel criteria, we assessed the performance of Nanopore-based SMS for the characterization of vaginal microbiomes in direct comparison to an established Illumina 16S-based approach. As a first step, we confirmed that the vaginal microbiomes of our study participants were broadly consistent with published vaginal microbiome structures of women of European ancestry [3], as well as with expected associations between the vaginal microbiome and BV [68].

The comparison between Nanopore-based shallow SMS and Illumina 16S showed that the two approaches were highly concordant with respect to the detection of dominant species and with respect to determining the CST of individual samples. We also observed a high degree of concordance with respect to the overall clustering structure (i.e., as observed in PCoA) of the characterized microbiomes. Carrying out a species-level comparison, we observed significant differences in the estimated relative abundances of individual taxa, most of which, however, had low overall abundances. The observed species-level differences could therefore be understood to mostly affect the fine-scale characterization of the sequenced microbiomes; in general, the observation of differences between SMS- and 16S-based approaches is to be expected and was also reported for other human microbiomes [70–72]. One particularly noteworthy difference between the two technologies concerned an increased abundance of *G. vaginalis* in the Nanopore data, also associated with an increase in the number of samples that were assigned to CST IV. Due to their amplification-free nature, SMS-based approaches can generally be expected to provide a more faithful representation of biological microbiome structures; the observed increased abundance of *G. vaginalis* could thus be interpreted as increased sensitivity of Nanopore-based shallow SMS for the detection of *Gardnerella*-driven dysbiosis of the vaginal microbiome [73].

In addition to characterizing the prokaryotic microbiome, SMS-based approaches may also enable insights into eukaryotic and viral microbiome components. We demonstrated the successful detection of *Candida albicans* and of 2 viral species (*Lactobacillus phage Lv-1*, *Human gammaherpesvirus 4*). Of note, the presence of a *Lactobacillus*-infecting phage in vaginal microbiomes is highly plausible and also supported by the literature [74–76], and the two detections of *Candida* were also supported by the microscopical detection of *Candida* (no fungal species were metagenomically detected in 6 other individuals with microscopical detection of *Candida*). Due to low overall read counts for non-human eukaryotic and viral taxa, our analyses of the non-prokaryotic microbiome should be considered exploratory in nature. It is likely that additional *Lactobacillus* phages and, potentially, eukaryotic species would have been detected at a higher sequencing depth. We also demonstrated the successful application of a methylation-based cell type deconvolution approach to the Nanopore shallow SMS data. The used methylation atlas does not include cell types from the female genital tract; analyses involving such input will therefore return results that best match available tissues in the database. The detection of prostate cells may be driven by epigenetic features shared with vaginal epithelial cells; to test this we carried out an exploratory analysis of average methylation values for 175 CpG sites hypermethylated in vaginal secretions [77] with our tissue deconvolution algorithm and obtained an estimated composition of 71% prostate cells (data not shown). Epigenetic similarities between vaginal and prostate cells may be associated with regulation by sex hormones; the detection of “head and neck laryngeal” cells may reflect epigenetic features associated with mucosal tissues (note that the label “head and neck laryngeal” is derived from the tissue atlas [65], with no further specification of the comprised cell types). No difference, however, was observed between the estimated tissue compositions of the BV and non-BV groups; similarly, we did not observe a difference between these groups with respect to the proportion of human reads.

One potential disadvantage of the Nanopore-based shallow SMS approach evaluated here that may preclude its application in larger-scale research or diagnostic settings is the observed variability in sequencing depths; despite employing a very lenient threshold of only 200 prokaryotic reads per sample, 2 samples had to be excluded from further analyses (in addition to the 4 samples that were excluded from all analyses due to unsuccessful DNA extraction). Characterizing the factors that influence per-sample sequencing depths could be an important direction for future research; these may include DNA quality, which was not measured in this study, but which could be the target of protocol optimization steps that lead to more predictable sequencing yields. Nanopore’s adaptive sampling enables selective human DNA depletion, as shown to increase total sequencing depth and enhance taxonomic sensitivity in vaginal samples [78]. While not used in our study, this approach holds promise for future applications.

In addition to analytical performance, the choice of sequencing technology is also affected by criteria such as input requirements, ease of use, scalability, and sequencing costs. While these criteria are affected by a variety of factors, including the specific processes used in the wet-lab and dynamic market conditions, we carried out a comparison between Illumina 16S and Nanopore shallow SMS along relevant dimensions (Supplementary table S15). While necessarily approximate and specific to the assumed use case, the comparison suggests that Nanopore SMS is particularly suited for analyzing up to 48 samples.

Limitations of our study include its limited size and the limited ethnic diversity of study participants; limited ethnic diversity may be of particular relevance due to the described associations between ethnic background and vaginal microbiome composition [3]. Nugent scores in the BV group did not in every case match with the clinically achieved Amsel criteria, which resulted in a mean Nugent score < 7 in the BV group. Despite including species-level analyses, our study was mostly focused on comparing Nanopore-based shallow SMS and Illumina 16S at the level of large-scale vaginal microbiome structures. Finally, our study was based on a relative comparison of two technologies, and we did not attempt to benchmark these technologies against a biological ground truth.

## Conclusions

In conclusion, our study demonstrated the successful application of Nanopore-based shallow SMS for the characterization of vaginal microbiomes, demonstrating a high degree of concordance between Nanopore-based shallow SMS and an established Illumina 16S sequencing protocol. We also demonstrated the Nanopore-based detection of features of the vaginal microbiome that are of potential diagnostic and biological importance and that are not accessible to 16S sequencing-based approaches. Our study therefore paves the way for the larger-scale application of Nanopore-based shallow SMS in larger-scale research or diagnostic settings, which may contribute to an improved understanding of incompletely understood important vaginal phenotypes, such as BV [20].

## Supplementary Information

Supplementary Material 1: S5: Sequencing statistics. Boxplots comparing BV and non-BV cohorts for: (A) DNA concentration, (B) 16S Illumina sequencing depth, and (C) SMS sequencing depth. Overlaid jitter plots represent individual measurements. S7: Rarefaction analysis of Illumina 16S data. Correlation between Shannon index values before and after rarefaction at a depth of 140,000 reads. S9: Correlation of single species abundances between in 16S and SMS data. Correlation of Lactobacillus species and G. vaginalis abundances in 16S and SMS samples. Respective Pearson´s r and p-value of the correlation line is displayed in the top left corner. S10: PCoA plot of shallow SMS-based bacterial relative abundances. Principal Coordinates Analysis (PCoA) based on Nanopore SMS sequencing and Bray-Curtis inter-sample distances. Dots are colored according to BV status (dark blue = BV; light blue = non-BV). Also shown is a contour plot of the probability density function (KDE) in dashed lines, which represent areas of equal probability density. S12: Visual inspection of genome coverage for non-human eukaryotic and viral species in Nanopore SMS data. Examples of read distributions. (A) examples of verified species, (B) examples of species that failed verification. Note that some reads and contigs appear black because only their borders are visible due to scaling. S13: Per-sample coverage on the human genome. Bar plot showing per-sample coverage of human genome. Red horizontal line represents a utilized threshold of 0.2X coverage. S2: SMS custom database content. An overview and detailed listing of the Kraken2/Bracken custom database content. The first sheet (“db_content_overview”) summarizes the number of assemblies and species per RefSeq division. The second sheet (“db_content_detailed”) provides genome-level details, including FTP download paths, release dates, species names, taxonomic IDs, and RefSeq divisions. S3: Study cohort metadata. A categorical overview of metadata of study participants (age, ethnicity, parity, BMI, vaginal pH, additional diagnoses, microscopy). S4: Sequencing statistics. Quantitative overview of DNA extraction (DNA concentration, number of extraction attempts), 16S and shallow SMS sequencing (sequencing depth, classification rate, broad composition). S6: 16S Illumina relative bacterial abundances. Relative bacterial abundances across samples. Rows represent individual samples, and columns correspond to bacterial species. For each sample, only species with relative abundances exceeding 5% were reported individually. Species with relative abundances below this threshold were aggregated and reported collectively under the category “Other”. Additionally, sequencing depth and community state type, derived from the bacterial composition, are reported for each sample. S8: Nanopore SMS relative bacterial abundances. Relative bacterial abundances across samples. Rows represent individual samples, and columns correspond to bacterial species. For each sample, only species with relative abundances exceeding 5% were reported individually. Species with relative abundances below this threshold were aggregated and reported collectively under the category “Other”. Additionally, sequencing depth, number of bacterial reads, and community state type, derived from the bacterial composition, are reported for each sample. Two samples were excluded from further analysis due to low read count. S11: Overview of reported and verified non-human eukaryotic and viral species in Nanopore SMS data. The overview includes sample information, detected species, taxonomic IDs, as well as back-mapping, alignment, and BLAST statistics. S14: Human cell type composition of samples based on a tissue deconvolution approach. The table includes human cell type composition and p-values from the Mann-Whitney U test for each cell type, comparing control and vaginosis groups. S15: Comparison between Illumina 16S and Nanopore shallow SMS sequencing approach. Comparison of input requirements, scalability, and estimated sequencing costs for 4 different multiplexing scenarios.

## Data Availability

Sequencing data that support the findings of this study (with human reads removed) have been deposited in NCBI SRA and can be accessed with the BioProject identifier PRJNA1229860.

## Abbreviations

BMI: Body mass index
BV: Bacterial vaginosis
CST: Community state type
PCoA: Principal coordinates analysis
PCR: Polymerase chain reaction
SFB: Short fragment buffer
SMS: Shotgun metagenomic sequencing
