# Supplementary figures and images for: Shallow shotgun metagenomic sequencing of vaginal microbiomes with the Oxford Nanopore technology enables the reliable determination of vaginal community state types and broad community structures
Source: BMC Microbiol. 2025 Aug 25;25:544. doi: 10.1186/s12866-025-04236-5 (PMC12376446; doi:10.1186/s12866-025-04236-5)

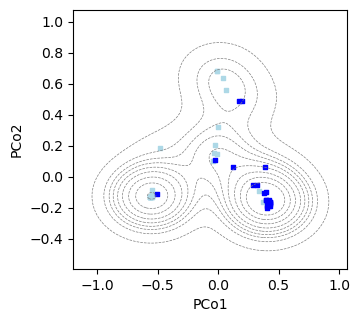

Supplement: Supplementary file 1 — Supplementary Material 1: S5: Sequencing statistics. Boxplots comparing BV and non-BV cohorts for: (A) DNA concentration, (B) 16S Illumina sequencing depth, and (C) SMS sequencing depth. Overlaid jitter plots represent individual measurements. S7: Rarefaction analysis of Illumina 16S data. Correlation between Shannon index values before and after rarefaction at a depth of 140,000 reads. S9: Correlation of single species abundances between in 16S and SMS data. Correlation of Lactobacillus species and G. vaginalis abundances in 16S and SMS samples. Respective Pearson´s r and p-value of the correlation line is displayed in the top left corner. S10: PCoA plot of shallow SMS-based bacterial relative abundances. Principal Coordinates Analysis (PCoA) based on Nanopore SMS sequencing and Bray-Curtis inter-sample distances. Dots are colored according to BV status (dark blue = BV; light blue = non-BV). Also shown is a contour plot of the probability density function (KDE) in dashed lines, which represent areas of equal probability density. S12: Visual inspection of genome coverage for non-human eukaryotic and viral species in Nanopore SMS data. Examples of read distributions. (A) examples of verified species, (B) examples of species that failed verification. Note that some reads and contigs appear black because only their borders are visible due to scaling. S13: Per-sample coverage on the human genome. Bar plot showing per-sample coverage of human genome. Red horizontal line represents a utilized threshold of 0.2X coverage. S2: SMS custom database content. An overview and detailed listing of the Kraken2/Bracken custom database content. The first sheet (“db_content_overview”) summarizes the number of assemblies and species per RefSeq division. The second sheet (“db_content_detailed”) provides genome-level details, including FTP download paths, release dates, species names, taxonomic IDs, and RefSeq divisions. S3: Study cohort metadata. A categorical overview of metadata of stu [file 12866_2025_4236_MOESM1_ESM.zip › Supplementary_material/S10_sSMS_PCoA.png]

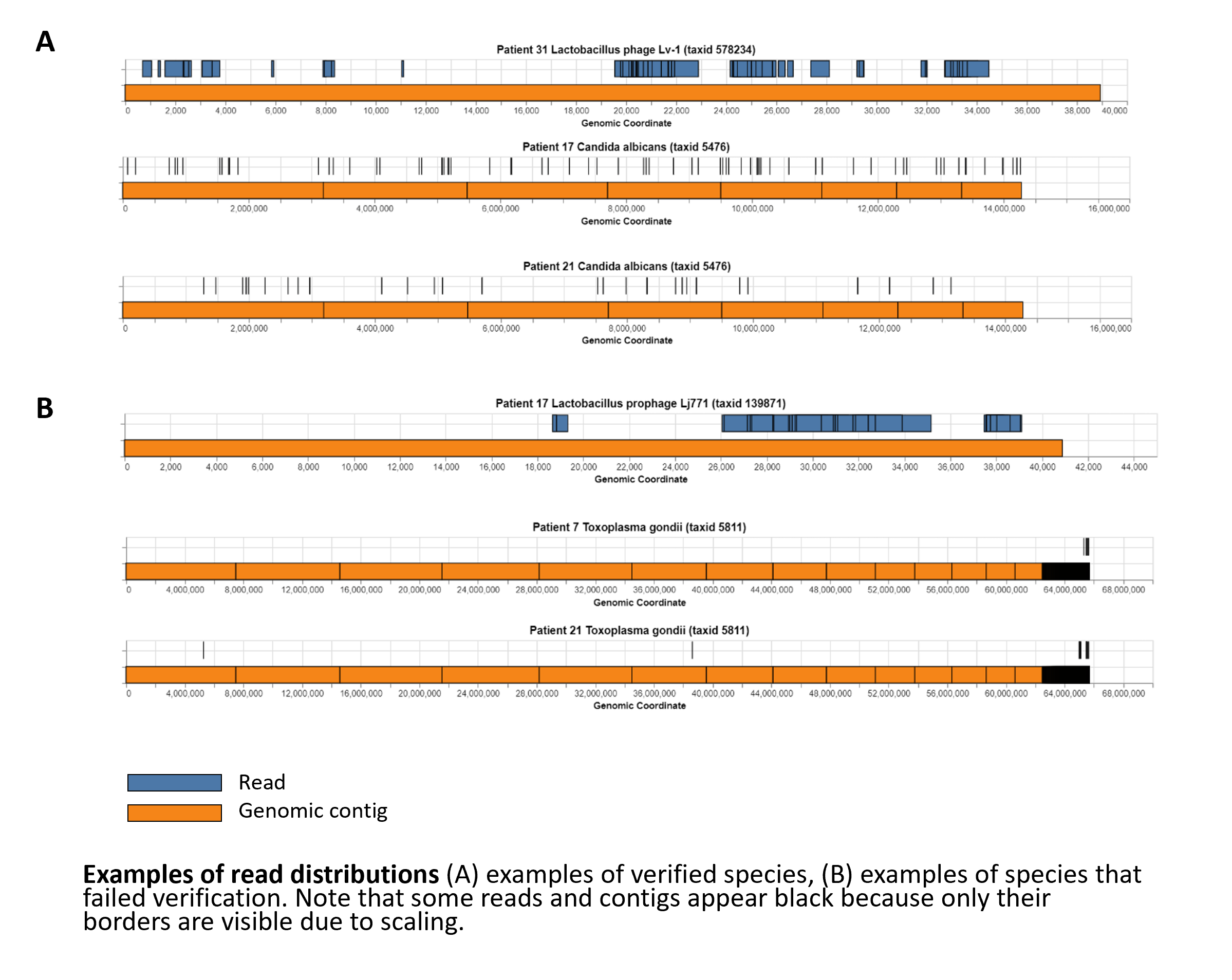

Supplement: Supplementary file 1 — Supplementary Material 1: S5: Sequencing statistics. Boxplots comparing BV and non-BV cohorts for: (A) DNA concentration, (B) 16S Illumina sequencing depth, and (C) SMS sequencing depth. Overlaid jitter plots represent individual measurements. S7: Rarefaction analysis of Illumina 16S data. Correlation between Shannon index values before and after rarefaction at a depth of 140,000 reads. S9: Correlation of single species abundances between in 16S and SMS data. Correlation of Lactobacillus species and G. vaginalis abundances in 16S and SMS samples. Respective Pearson´s r and p-value of the correlation line is displayed in the top left corner. S10: PCoA plot of shallow SMS-based bacterial relative abundances. Principal Coordinates Analysis (PCoA) based on Nanopore SMS sequencing and Bray-Curtis inter-sample distances. Dots are colored according to BV status (dark blue = BV; light blue = non-BV). Also shown is a contour plot of the probability density function (KDE) in dashed lines, which represent areas of equal probability density. S12: Visual inspection of genome coverage for non-human eukaryotic and viral species in Nanopore SMS data. Examples of read distributions. (A) examples of verified species, (B) examples of species that failed verification. Note that some reads and contigs appear black because only their borders are visible due to scaling. S13: Per-sample coverage on the human genome. Bar plot showing per-sample coverage of human genome. Red horizontal line represents a utilized threshold of 0.2X coverage. S2: SMS custom database content. An overview and detailed listing of the Kraken2/Bracken custom database content. The first sheet (“db_content_overview”) summarizes the number of assemblies and species per RefSeq division. The second sheet (“db_content_detailed”) provides genome-level details, including FTP download paths, release dates, species names, taxonomic IDs, and RefSeq divisions. S3: Study cohort metadata. A categorical overview of metadata of stu [file 12866_2025_4236_MOESM1_ESM.zip › Supplementary_material/S12_visual_inspection_nonbact.png]

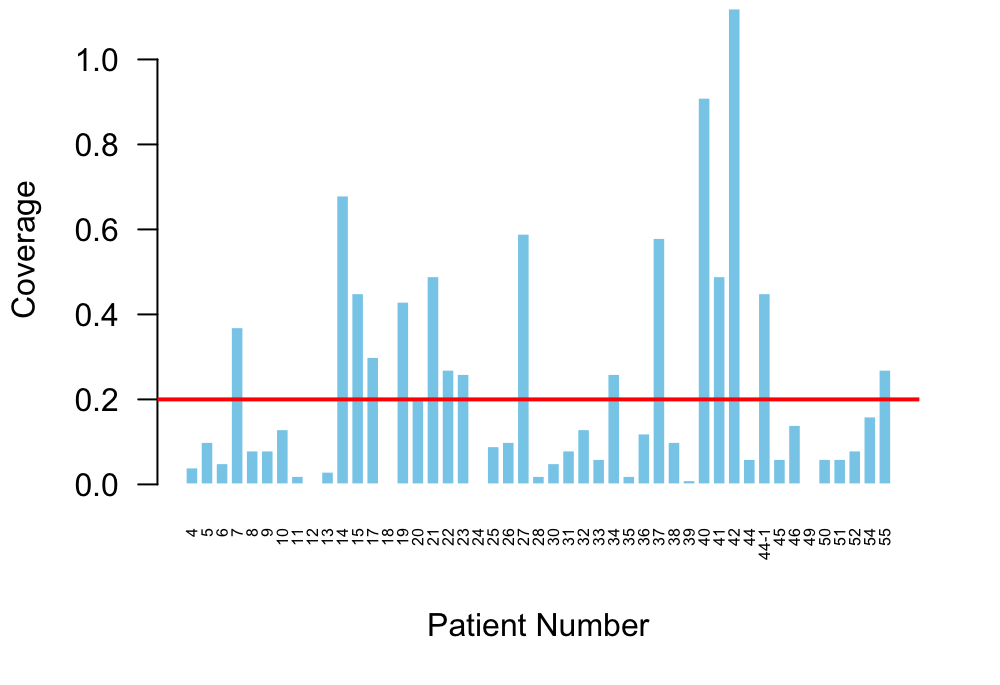

Supplement: Supplementary file 1 — Supplementary Material 1: S5: Sequencing statistics. Boxplots comparing BV and non-BV cohorts for: (A) DNA concentration, (B) 16S Illumina sequencing depth, and (C) SMS sequencing depth. Overlaid jitter plots represent individual measurements. S7: Rarefaction analysis of Illumina 16S data. Correlation between Shannon index values before and after rarefaction at a depth of 140,000 reads. S9: Correlation of single species abundances between in 16S and SMS data. Correlation of Lactobacillus species and G. vaginalis abundances in 16S and SMS samples. Respective Pearson´s r and p-value of the correlation line is displayed in the top left corner. S10: PCoA plot of shallow SMS-based bacterial relative abundances. Principal Coordinates Analysis (PCoA) based on Nanopore SMS sequencing and Bray-Curtis inter-sample distances. Dots are colored according to BV status (dark blue = BV; light blue = non-BV). Also shown is a contour plot of the probability density function (KDE) in dashed lines, which represent areas of equal probability density. S12: Visual inspection of genome coverage for non-human eukaryotic and viral species in Nanopore SMS data. Examples of read distributions. (A) examples of verified species, (B) examples of species that failed verification. Note that some reads and contigs appear black because only their borders are visible due to scaling. S13: Per-sample coverage on the human genome. Bar plot showing per-sample coverage of human genome. Red horizontal line represents a utilized threshold of 0.2X coverage. S2: SMS custom database content. An overview and detailed listing of the Kraken2/Bracken custom database content. The first sheet (“db_content_overview”) summarizes the number of assemblies and species per RefSeq division. The second sheet (“db_content_detailed”) provides genome-level details, including FTP download paths, release dates, species names, taxonomic IDs, and RefSeq divisions. S3: Study cohort metadata. A categorical overview of metadata of stu [file 12866_2025_4236_MOESM1_ESM.zip › Supplementary_material/S13_Per_sample_human_read_coverage.png]

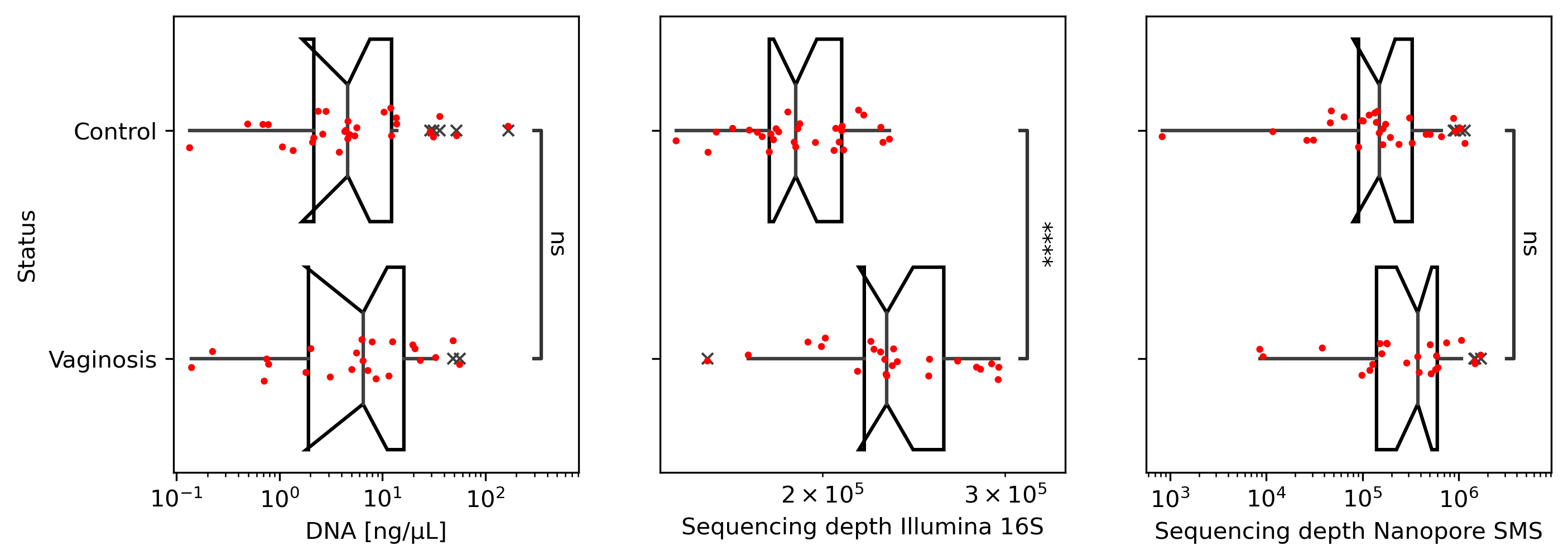

Supplement: Supplementary file 1 — Supplementary Material 1: S5: Sequencing statistics. Boxplots comparing BV and non-BV cohorts for: (A) DNA concentration, (B) 16S Illumina sequencing depth, and (C) SMS sequencing depth. Overlaid jitter plots represent individual measurements. S7: Rarefaction analysis of Illumina 16S data. Correlation between Shannon index values before and after rarefaction at a depth of 140,000 reads. S9: Correlation of single species abundances between in 16S and SMS data. Correlation of Lactobacillus species and G. vaginalis abundances in 16S and SMS samples. Respective Pearson´s r and p-value of the correlation line is displayed in the top left corner. S10: PCoA plot of shallow SMS-based bacterial relative abundances. Principal Coordinates Analysis (PCoA) based on Nanopore SMS sequencing and Bray-Curtis inter-sample distances. Dots are colored according to BV status (dark blue = BV; light blue = non-BV). Also shown is a contour plot of the probability density function (KDE) in dashed lines, which represent areas of equal probability density. S12: Visual inspection of genome coverage for non-human eukaryotic and viral species in Nanopore SMS data. Examples of read distributions. (A) examples of verified species, (B) examples of species that failed verification. Note that some reads and contigs appear black because only their borders are visible due to scaling. S13: Per-sample coverage on the human genome. Bar plot showing per-sample coverage of human genome. Red horizontal line represents a utilized threshold of 0.2X coverage. S2: SMS custom database content. An overview and detailed listing of the Kraken2/Bracken custom database content. The first sheet (“db_content_overview”) summarizes the number of assemblies and species per RefSeq division. The second sheet (“db_content_detailed”) provides genome-level details, including FTP download paths, release dates, species names, taxonomic IDs, and RefSeq divisions. S3: Study cohort metadata. A categorical overview of metadata of stu [file 12866_2025_4236_MOESM1_ESM.zip › Supplementary_material/S5_sequencing_statistics.png]

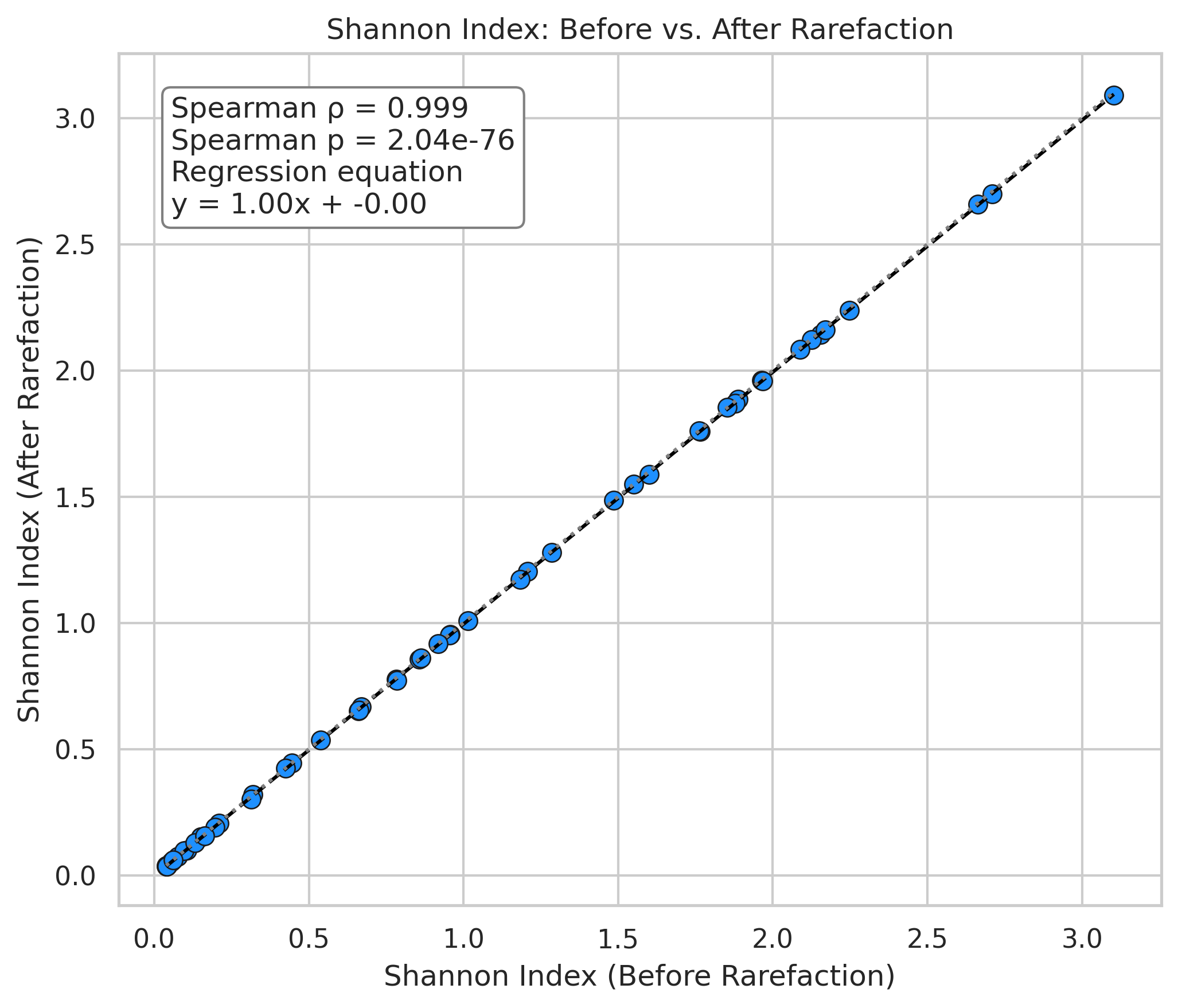

Supplement: Supplementary file 1 — Supplementary Material 1: S5: Sequencing statistics. Boxplots comparing BV and non-BV cohorts for: (A) DNA concentration, (B) 16S Illumina sequencing depth, and (C) SMS sequencing depth. Overlaid jitter plots represent individual measurements. S7: Rarefaction analysis of Illumina 16S data. Correlation between Shannon index values before and after rarefaction at a depth of 140,000 reads. S9: Correlation of single species abundances between in 16S and SMS data. Correlation of Lactobacillus species and G. vaginalis abundances in 16S and SMS samples. Respective Pearson´s r and p-value of the correlation line is displayed in the top left corner. S10: PCoA plot of shallow SMS-based bacterial relative abundances. Principal Coordinates Analysis (PCoA) based on Nanopore SMS sequencing and Bray-Curtis inter-sample distances. Dots are colored according to BV status (dark blue = BV; light blue = non-BV). Also shown is a contour plot of the probability density function (KDE) in dashed lines, which represent areas of equal probability density. S12: Visual inspection of genome coverage for non-human eukaryotic and viral species in Nanopore SMS data. Examples of read distributions. (A) examples of verified species, (B) examples of species that failed verification. Note that some reads and contigs appear black because only their borders are visible due to scaling. S13: Per-sample coverage on the human genome. Bar plot showing per-sample coverage of human genome. Red horizontal line represents a utilized threshold of 0.2X coverage. S2: SMS custom database content. An overview and detailed listing of the Kraken2/Bracken custom database content. The first sheet (“db_content_overview”) summarizes the number of assemblies and species per RefSeq division. The second sheet (“db_content_detailed”) provides genome-level details, including FTP download paths, release dates, species names, taxonomic IDs, and RefSeq divisions. S3: Study cohort metadata. A categorical overview of metadata of stu [file 12866_2025_4236_MOESM1_ESM.zip › Supplementary_material/S7_rarefaction_Illumina_16S.png]

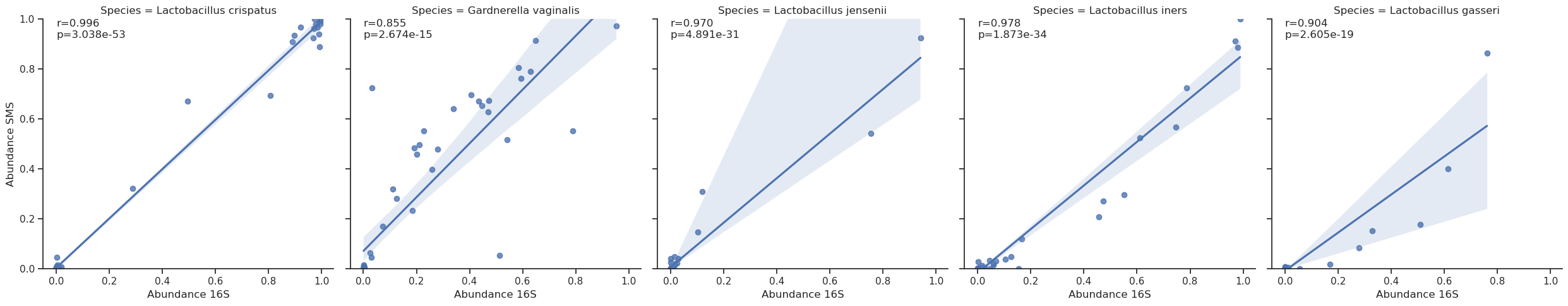

Supplement: Supplementary file 1 — Supplementary Material 1: S5: Sequencing statistics. Boxplots comparing BV and non-BV cohorts for: (A) DNA concentration, (B) 16S Illumina sequencing depth, and (C) SMS sequencing depth. Overlaid jitter plots represent individual measurements. S7: Rarefaction analysis of Illumina 16S data. Correlation between Shannon index values before and after rarefaction at a depth of 140,000 reads. S9: Correlation of single species abundances between in 16S and SMS data. Correlation of Lactobacillus species and G. vaginalis abundances in 16S and SMS samples. Respective Pearson´s r and p-value of the correlation line is displayed in the top left corner. S10: PCoA plot of shallow SMS-based bacterial relative abundances. Principal Coordinates Analysis (PCoA) based on Nanopore SMS sequencing and Bray-Curtis inter-sample distances. Dots are colored according to BV status (dark blue = BV; light blue = non-BV). Also shown is a contour plot of the probability density function (KDE) in dashed lines, which represent areas of equal probability density. S12: Visual inspection of genome coverage for non-human eukaryotic and viral species in Nanopore SMS data. Examples of read distributions. (A) examples of verified species, (B) examples of species that failed verification. Note that some reads and contigs appear black because only their borders are visible due to scaling. S13: Per-sample coverage on the human genome. Bar plot showing per-sample coverage of human genome. Red horizontal line represents a utilized threshold of 0.2X coverage. S2: SMS custom database content. An overview and detailed listing of the Kraken2/Bracken custom database content. The first sheet (“db_content_overview”) summarizes the number of assemblies and species per RefSeq division. The second sheet (“db_content_detailed”) provides genome-level details, including FTP download paths, release dates, species names, taxonomic IDs, and RefSeq divisions. S3: Study cohort metadata. A categorical overview of metadata of stu [file 12866_2025_4236_MOESM1_ESM.zip › Supplementary_material/S9_correlation_lactobacillus.png]
